# Tunable magnetocaloric effect in transition metal alloys

**DOI:** 10.1038/srep15755

**Published:** 2015-10-28

**Authors:** Dustin D. Belyea, M. S. Lucas, E. Michel, J. Horwath, Casey W. Miller

**Affiliations:** 1 Department of Physics, University of South Florida, 4202 East Fowler Ave, Tampa, FL 33620; 2 Air Force Research Laboratory, Wright-Patterson AFB, Ohio 45433, USA; 3 UTC, Inc., 1270 North Fairfield Road, Dayton, OH 45432, USA; 4 Wright State University, Dayton, OH 45435, USA; 5 School of Chemistry and Materials Science, Rochester Institute of Technology, 85 Lomb Memorial Drive, Rochester, NY 14623.

## Abstract

The unpredictability of geopolitical tensions and resulting supply chain and pricing instabilities make it imperative to explore rare earth free magnetic materials. As such, we have investigated fully transition metal based “high entropy alloys” in the context of the magnetocaloric effect. We find the NiFeCoCrPd*x* family exhibits a second order magnetic phase transition whose critical temperature is tunable from 100 K to well above room temperature. The system notably displays changes in the functionality of the magnetic entropy change depending on *x*, which leads to nearly 40% enhancement of the refrigerant capacity. A detailed statistical analysis of the universal scaling behavior provides direct evidence that heat treatment and Pd additions reduce the distribution of exchange energies in the system, leading to a more magnetically homogeneous alloy. The general implications of this work are that the parent NiFeCoCr compound can be tuned dramatically with FCC metal additives. Together with their relatively lower cost, their superior mechanical properties that aid manufacturability and their relative chemical inertness that aids product longevity, NiFeCoCr-based materials could ultimately lead to commercially viable magnetic refrigerants.

The magnetocaloric effect (MCE) is a thermodynamic phenomenon being exploited for the next generation of highly efficient refrigeration technologies. The MCE enables a refrigerant's temperature to change with applied magnetic fields^1^,^2^,^3^,^4^,^5^,^6^, which allows for a simple refrigeration cycle. As in adiabatic demagnetization, which is used to reach microKelvin temperatures in solids, changes in a material's magnetic entropy 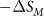 through an adiabatic process results in a change in the lattice entropy, and thus a temperature change. Since the discovery of the giant MCE in certain Gd silicates^7^,^8^, MCE research has led to the potential for magnetic refrigeration in room temperature applications with efficiencies of up to 60% of the Carnot limit^9^. While the magnetocaloric effect is generally large for rare earth metals, geopolitical issues and their related price fluctuations have catalyzed the exploration of transition metal systems with potential for magnetocaloric applications^10^,^11^. Further, though magnetocaloric materials exhibiting first order transitions are scientifically en vogue because they have higher entropy changes than most second order materials, they suffer major disadvantages for commercial viability. In particular, materials with coupled magneto-structural transitions suffer from cracking and fatigue, which severely limits their useful lifetime. Together, these points suggest it is important to explore transition metal systems displaying second order transitions for technological applications, since these systems offer supply chain and cost stability, and superior mechanical properties such as ductility, corrosion resistance, machinability, all of which ease manufacturing and bolster product longevity.

The so-called “high entropy alloys” (HEAs) are a class of emergent transition metal alloys that hold great potential for advanced manufacturing, and which may impact magnetocalorics. These are near equimolar alloys with high entropy of mixing when in a random solid solution. These materials have been pursued for high hardness and resistance to wear and corrosion, which are attractive properties for a myriad of applications^12^,^13^. Fe_1_Co_1_Ni_1_Cr_1_Pd_*x*_ is an example HEA system with tunable magnetic properties. The addition of Pd was shown to change the critical temperature (*T*_*c*_) from 300 to 500 K for *x* = 1 to 2, along with enhancement of the saturation magnetization^14^; previous works have used high percentages of Pd as a stabilizing agent in similar high strength bulk alloys^15^. In this work, we explore the magnetic properties of the Fe_1_Co_1_Ni_1_Cr_1_Pd_*x*_ alloy family, as-rolled and after annealing, with low molar fractions of Pd. We find improvement in the magnetocaloric properties throughout the system. A detailed statistical analysis of the universal scaling behavior suggest this originates predominately from the homogenization of the alloy with both Pd and heat treatment. While the use of Pd is disadvantageous because of its cost, we find benefit from as little as 3 atomic percent of this model additive. An important implication of this work is that other FCC metals, e.g., Cu or Ag, may have similar potential to tune the magnetism of this system, which would significantly reduce the materials cost, thereby bolstering commerical potential.

## Methods

The FeCoNiCrPd_*x*_ samples were made using an arc furnace under argon atmosphere using elemental Fe, Co, Cr, Ni, and Pd all of 99.99% or greater purity. Melted buttons were then cold rolled into thin sheets with thickness of 100 to 250 *μ*m, then diced to have areas of 2 mm × 3 mm. The samples were characterized before and after a heat treatment procedure, during which they were wrapped in Ta foil, sealed in a quartz tube with Ar gas, and annealed for one hour at 900 °C. The sample stoichiometries range from Fe_1_Co_1_Ni_1_Cr_1_Pd_0_ to Fe_1_Co_1_Ni_1_Cr_1_Pd_0.5_, or as atomic percentages of Pd: 0.00% (x = 0), 0.12%, 0.74%, 1.48%, 2.91%, 5.88% and 11.11% (x = 0.50). Magnetic characterization was performed on samples using a Quantum Design PPMS with a VSM option. *M*(*H*) is measured with fields from 0 to 50 kOe (stopping to measure at each field with the magnet in persistent mode) in appropriate step sizes. These quarter-*M*(*H*) loops are measured for isotherms ranging from 10 K to 350 K in steps of 5 K. This is important as it gives discrete M(H,T) data which is necessary for the accurate numerical calculation of −Δ*S* from Maxwell relations:


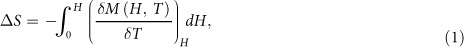


where we have dropped the subscript *M* on the entropy symbol, since we only concern ourselves in this paper with magnetic entropy. Due to the dependence of −Δ*S*(Δ*H*, *T*) on the derivative of the *M*(*T*), this allows −Δ*S* to serve as a probe into fundamental aspects of magnetic materials outside the research into magnetic refrigeration^16^,^17^. Separately, *M*(*T*) for low field and *M*(*H*) for low temperature measurements were taken to characterize basic magnetic properties of the materials. The *T*_*C*_ was estimated by the temperature at which *dM*/*dT* has a maximum value in a constant field of 2 kOe. X-ray Diffraction data were collected via standard *θ*–2*θ* configuration using Cu *K*_*α*_ photons.

## Results

### Magnetic Properties

**Figure 1:**
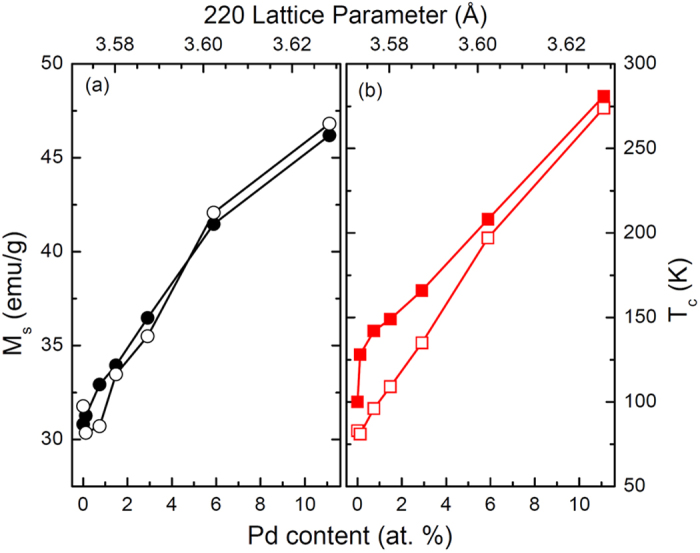
(**a**) Saturation magnetization from *M*(*H*) loops at 5 K (circles), and (**b**) Curie temperature from *M*(*T*) in 2 kOe (squares) in the as-rolled (closed) and annealed (open) states for NiFeCoCr_1_Pd_*x*_ as functions of Pd content and (220) lattice parameter.

All alloys studied displayed second order magnetic phase transitions. The *M*(*T*) and *M*(*H*) data yield measures of *T*_*c*_ and saturation magnetization (*M*_*s*_) as a function of Pd content for the materials in both the as-rolled state and after the annealing process (Fig. 1). Both *M*_*s*_ and *T*_*c*_ increase with the inclusion of Pd with similar scaling for both the as-rolled and annealed states. The values of *M*_*s*_ at 5 K and *T*_*c*_ were approximately 30 emu/g and 100 K for the 0% composition, and increased monotonically with Pd content to approximately 46 emu/g and 280 K, respectively, for the 11.11% composition. The enhancement of *M*_*s*_ and *T*_*c*_ with Pd incorporation is a straightforward consequence of mean field theory and the Bethe-Slater curve, which tells us that increasing the lattice parameter increases the exchange interaction and concomitant magnetic properties^18^; the X-Ray Diffraction (XRD) data below support this. Since *T*_*c*_ generally defines the mid point of a refrigerant's useful temperature range^19^, this system has the potential to provide tunable operating temperatures from 100 K to 300 K.

### X-Ray Diffraction

A previous study on NiFeCoCr_*x*_ showed that the cold rolling process affects the magnetic properties, where the as-rolled material has a re-entrant magnetic phase upon heating that is not present after heating to 800 °C^20^. The heat treatment had noticeable effects on the XRD patterns, where there was a sharpening of the peaks and changes in their relative intensities. This was interpreted as a reduction in the imperfections of the as-rolled material. Similar XRD measurements were performed in this study both before and after a 900 °C heat treatment for all samples and during heating for the NiFeCoCr sample.

**Figure 2:**
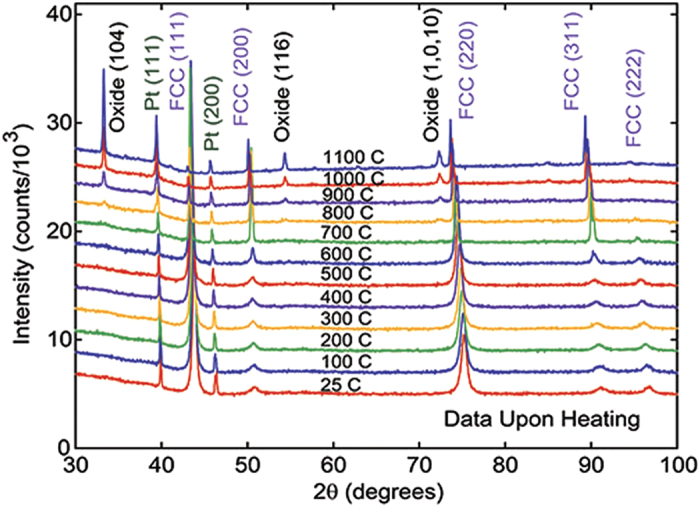
Temperature dependent wide angle XRD for x = 0.00% reveals a structural transition between 600 and 700 °C.

Figure 2 shows wide angle scans on the x = 0.00% sample from room temperature in the as-rolled state to 1100 °C. Between 600 and 700 °C there is a decrease in the full width at half maximum (FWHM) of the FCC NiFeCoCr peaks and a simultaneous change in the relative intensities of the indexed peaks; the (111), (222), and (311) peaks decrease in relative intensity while the (311) peak increases. Around 800 °C the presence of an oxide phase is observed along with a reduction in the FCC phase. This phase resembles the Cr_2_O_3_ phase Eskolaite (PDF card number 01-082-1484)^21^ but with (107) preferred orientation with a March coefficient of 0.271 at 1000 °C. Also at 1000 °C, The FCC NiFeCoCr peaks needed to be fit to two different FCC cards (a = 3.630 Å), one with (411) preferred orientation and a March coefficient of 0.228 and the other with (220) preferred orientation and a March coefficient of 0.113. After cooling from 1100 °C the oxide phase dominated the XRD pattern, though very sharp FCC peaks were observed.

**Figure 3:**
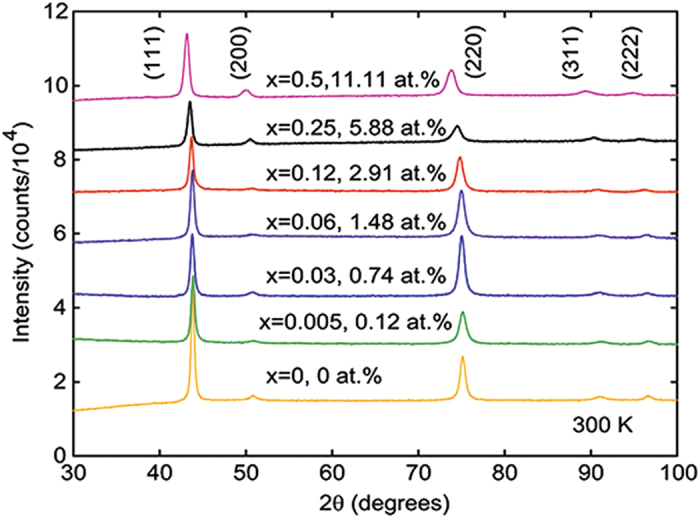
Room temperature diffraction data reveal the impact of Pd is to increase the lattice parameter; the increased peak breadth is significantly reduced with annealing.

All of the as-rolled samples had XRD patterns that were similar in relative intensities to those reported for FeCoCr_*x*_Ni alloys^20^. As shown in Fig. 3, the effect of Pd additions is to increase the lattice parameters. The cold rolled samples also showed a trend of increasing FWHM with Pd content. After heat treatment at 900 °C, the samples all had reduced FWHM, similar to the *in-situ* pattern for the NiFeCoCr sample above 700 °C. This implies an increase in structural coherence, which will be manifested as a tighter distribution of exchange energies, and thus more homogeneous magnetic properties.

The XRD patterns indicate that heat treatment has a profound impact on the lattice strain and grain size, where annealing significantly reduces the FWHM of all peaks. As shown below, this corresponds to an increase in the magnetic homogeneity of the samples. A large FWHM may indicate strain and/or chemical inhomogeneities induced by the arc-melting and cold rolling process. The large FWHM could also indicate very small grains as one might see in a ball milling process. Our samples have been brutalized, a testament to their ductility and production worthiness, and as such there should be effects of strain, defects (both vacancies and stacking faults), and grain size reduction. All three of these effects would decrease with annealing leading to a sharpened diffraction peak. The addition of Pd has similar effects on the magnetic homogeneity as annealing. However, the broadening of the XRD peaks with Pd content does not appear consistent with this trend. It is possible that the damage done to the surface of the sample during cold-rolling is not indicative of the bulk and that Pd aids in reducing defects. In this case, a technique such as high resolution neutron scattering may provide more useful structural information, as this would be a probe of the bulk rather than the surface.

### Magnetocaloric Effect

**Figure 4:**
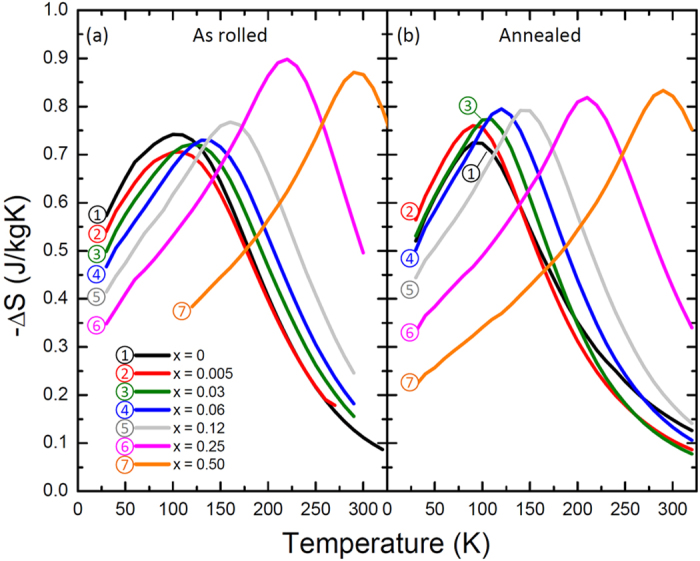
Magnetic entropy change with Δ*H* = 50 kOe (**a**) as rolled and (**b**) after annealing for NiFeCoCrPd_*x*_.

Figure 4 shows the magnetic entropy changes for all samples for Δ*H* = 50 kOe. For the as-rolled samples we find that Pd additions shift the temperature at which −Δ*S* peaks (*T*_*peak*_) from ≈100 K to 300 K, as expected from the *T*_*c*_ shift. This is accompanied by relatively small changes in −Δ*S*_*max*_ for increasing *x*. There is, however, a notable change in the functionality (shape) of the −Δ*S*(*T*) curves, specifically, increasing Pd content gives a decrease in the width and alters the overall peaked structure to have a carat shape more typical of magnetocaloric materials with second order phase transitions. This makes sense because the as-rolled samples will have the largest density of defects, and thus the largest distribution of exchange parameters, which in turn leads to inhomogeneous magnetic properties that are evidenced by the uncharacteristic functionality. Once the defect density is reduced by heat treatment, the materials trend toward the typical carat shape. It is also noteworthy that the incorporation of Pd even in the as-rolled state has an impact on the magnetic properties, as evidenced by the less rounded dS features for low Pd contents. This is in line with previous results that show the Pd tends to homogenize the structure^14^. We see a similar scaling in *T*_*peak*_ with *x* in the annealed samples, but the change in functionality of the −Δ*S* curves is no longer as distinctive (Fig. 4(b)).

Changes in −Δ*S*_*max*_ as well as the changes in the shape of the −Δ*S*(Δ*H*, *T*) curves are important to MCE when applied to magnetic refrigeration, as they may lead to changes in the overall refrigerant capacity (RC). RC in general is the figure of merit used to compare MCE materials giving the total heat transfer of the material over a typical refrigeration cycle. RC for a given Δ*H* is calculated as the integral of −Δ*S*(*T*) over a temperature range of interest^22^,^23^:


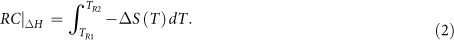


**Figure 5:**
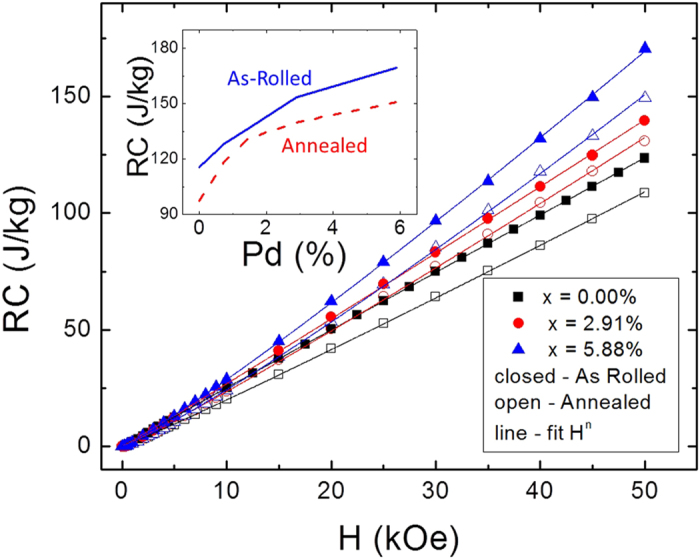
Refrigerant capacity of NiFeCoCr_1_Pd_*x*_ for select Pd contents: x = 0% (squares), x = 2.91% (circles) and x = 5.88% (triangles), in both the as-rolled (open) and annealed states (closed). Lines are power law fits. The insert shows RC for Δ*H* = 50 kOe for the series of samples under both heat treatments.

Here, T_*R*1_ and T_*R*2_ are traditionally taken to be the temperatures corresponding to the FWHM of the −Δ*S*(*T*) feature on the low and high temperature sides of the peak, respectively. We do not consider hysteretic losses here, as the M(H) loops showed negligible hysteresis, though that can be important in many systems^24^. Figure 5 shows RC as a function of Δ*H* for both the annealed and as-rolled samples. The RC of 36 J/kg for a 1 T field change puts these HEAs below many other materials designed to operate in the 100–200 K range^3^, though several factors related to pragmatic engineering issues (e.g., ductility, corrosion resistance, high thermal conductivity) may make up for this. The as-rolled samples have RC increased approximately 25% over the annealed samples, which is nearly independent of Δ*H*. This enhancement is likely artificial, i.e., a consequence of disorder broadening the distribution of exchange parameters, and thus broadening the apparent temperature breadth. While still actively under investigation, increased widths may be useful in graded or otherwise multi-component refrigerants, in which composition differences may enable distinct operation points^25^. These results show, nonetheless, that Pd incorporation leads to improved RC. The insert to Fig. 5 shows that, for a fixed Δ*H* of 50 kOe, RC is enhanced with Pd by nearly 50% and 60% in the as-rolled and annealed samples, respectively. For the latter, most of the increase (40%) is observed with less than 1.5% Pd. The reduction in RC with annealing is expected, as the M(T) data all show a sharper transition at *T*_*c*_, and thus a reduced FWHM of the entropy change peak.

It has been shown previously that the field dependence of −ΔS_*M*_ and RC can be related to the critical exponents^26^:


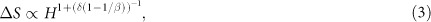



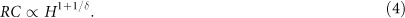


**Figure 6:**
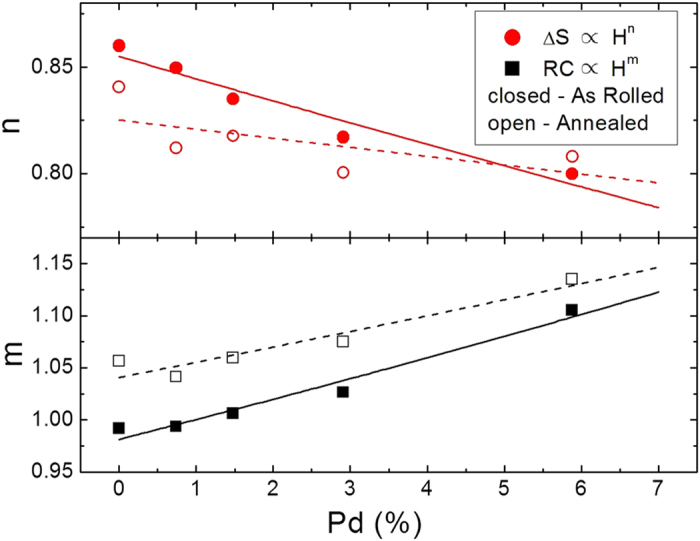
The power law exponents for the Δ*S*_*max*_ and RC behavior, *n* (circles) and *m* (squares), decrease and increase, respectively, with Pd content.

The behavior of the entropy change peak and refrigerant capacity with applied field were fit to power functions (Fig. 5), namely −Δ*S*_*max*_ ∝ H^*n*^ and RC ∝ H^*m*^; mean field theory predicts *n* = 2/3 and *m* = 4/3. We annotate these exponents with subscripts that describe the heat treatment (*a*nnealed or *u*nannealed) and Pd content. Figure 6 shows resulting values of *n* and *m* for all samples as a function of Pd atomic percent. For the entropy change, *n* is always sublinear, and decreases with both Pd and annealing: *n*_*u*,0%_ = 0.86 falls to *n*_*u*,5.88%_ = 0.81 with Pd added, while annealing reduces the exponent: *n*_*a*,0%_ = 0.84. The behavior of RC is nearly linear with field for the unannealed, x = 0 sample (*m*_*u*,0%_ = 0.99), but becomes superlinear with 6% Pd added (*m*_*u*,5.88%_ = 1.10). Annealing further increases *m*, with *m*_*a*,0%_ = 1.05 and *m*_*a*,5.88%_ = 1.13. These results imply that the improvement of the refrigerant capacity with Pd has less to do with overall magnitude of the entropy change and more with the increased susceptibility of the material near the phase transition. While additional Pd may lead to some further improvement, that improvement should become weaker as the lattice parameter moves the system toward the peak of the Bethe-Slater curve.

It is straightforward to compare the behavior of these exponents with mean field theory (values previously noted) and the 3D Ising model (*n* = 0.897 and *m* = 1.21)^6^. The observed changes in ΔS_*M*_ exponent with Pd content (Fig. 6) seems to favor the mean field model, decreasing toward that expected exponent, while moving further from the expectation for the 3D Ising model. Since the observed increase in RC exponent with annealing and Pd is in line with both models, we conclude mean field is more appropriate. The increase in Pd content thus gives the material the more prototypical response, which suggests it is more magnetically homogeneous. Neutron scattering could be employed to understanding this quantitatively. That mean field behavior seems to be appropriate for these alloys is significant. This may enable computational materials scientists to survey the large parameter space occupied by materials containing four or more elements.

### Universal Scaling Analysis Reveals Magnetic Homogenization

Universal scaling analysis quantitatively tells us that the Pd helps to homogenize the magnetic properties in these alloys^27^. This method should remove the temperature and field dependence of the set of Δ*S*(Δ*H*, *T*) curves (for fixed Δ*H*) so that all curves processed with the same scaling protocol collapse onto a single universal curve. A failure to display this universal collapse can be attributed to inhomogeneity within the material^28^, likely in the form of a distribution of exchange energies. As detailed below, we employ a sophisticated statistical analysis to quantify the degree to which the set of scaled curves collapse onto a universal curve.

**Figure 7:**
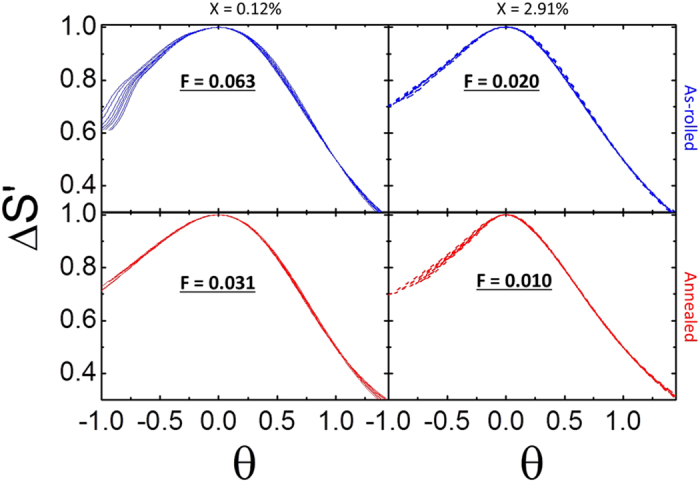
Scaled curves calculated from the ΔS_*M*_(ΔH,T) for the x = 0.12% (solid) and x = 2.91% (dashed) both as-rolled (blue) and annealed (red) for fields ranging from 15 to 50 kOe in steps of 5 kOe. The F values indicate the level of homogeneity within the set of ten curves in each plot. Annealing and adding Pd both increase the magnetic homogeneity of the system; the greatest improvement utilized both.

We scale the entropy axis by defining 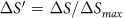; the scaled temperature axis is defined as:


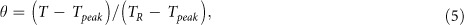


where *T*_*peak*_ is the temperature at Δ*S*_*max*_ for each field, and *T*_*R*_ is the temperature at 75% of Δ*S*_*max*_ above *T*_*peak*_. As shown in Fig. 7 for x = 0.12% and x = 2.91%, the curves do not collapse onto a single curve. It is apparent by visual inspection, though, that the degree of collapse increases when Pd is added. We can quantify the degree of collapse using statistical methods, namely the Brown-Forsythe analysis of variance (BF method). The BF method gives a single statistic, F, which directly measures the homogeneity of variance between a group of peaked curves. F is zero when a group of curves is identical, and increases from there as the set of curves becomes more inhomogeneous. This therefore gives us direct evidence that distributions of exchange interactions exist in the material: a small distribution will be essentially a perfect homogeneous material, and will have F approaching zero; a broad distribution will lead to increased F.

We observe a decrease in F of at least 50% with annealing or Pd incorporation, and an additional 50% decrease with both annealing and adding Pd content. These results are direct experimental evidence that both heat treatment and composition cause a homogenization of the magnetic properties. The F statistic was calculated only for the values of *θ* > 0 in order to compare all samples with the same protocol (i.e., the low Pd containing samples do not have sufficient data in the *θ* < 0 regime); a separate study we performed implies this has no bearing on the validity of the method, but it does impact the absolute magnitude of F.

## Discussion

Our results show there is a major impact on magnetic properties of this novel class of materials with FCC metal additions, even at small concentrations. The NiFeCoCrPd_*x*_ displays a second order magnetic phase transition that is tunable from room temperature down to around 100 K as *x* decreases from 11.11% down to 0.00%. While the magnetic entropy change for the transition only increases slightly with Pd, the refrigerant capacity improves by nearly 40%. The latter observation is related to increasingly superlinear behavior of the field dependence of the refrigerant capacity with Pd content. This increase in field dependence may be attributed to increased magnetic homogeneity within the alloy as a result of both Pd additions and annealing, which appear to reduce the distribution of exchange energies in the system. The initially wide distribution of exchange energies is at least the result of structural defects, and possibly due to an unidentified minority phase (as has been observed in other HEAs^14^). The increase in magnetic homogeneity was directly evidenced by an increase in the homogeneity of variance of universal scaling curves associated with the magnetic entropy change at the magnetic phase transition. In other words, our ability to quantitatively characterize the degree to which universal scaling works in the system helps us understand that the distribution of exchange interactions becomes narrower, thus resembling a single phase magnetic material, with Pd incorporation and heat treatment.

Since the drive for magnetic refrigeration is targeted around room temperature operation, it is important to discuss the high entropy alloys relative to gadolinium. The NiFeCoCrPd_0.5_ alloy has the appropriate critical point, but its maximum entropy change per field is about a factor of 20 less than that of Gd (about 3 J/kgK for a 1 T field change^27^). At the same time, the significant cost of Pd makes this alloy ten times as expensive as Gd. Thus, while this basic science study focused on an alloy with limited commercial potential, our findings strongly suggest that other nominally FCC metals such as Ag or Cu should lead to similar tunability of the HEAs, but with tremendously reduced materials cost. For example, the cost of NiFeCoCrCu_0.5_ is about 5% that of bulk Gd, and the cost of NiFeCoCrAg_0.5_ is about 41% of Gd. Should these alloys have roughly the same magnetic entropy change as the Pd-containing alloys, which is possible because their role is dominantly structural in nature, then HEAs may become serious contenders for commercial applications.

To put the high entropy alloys into context within the general class of magnetocaloric mateirals, we must consider that a particular material is deployed for a specific operating temperature range. For example, Gd is a poor magnetocaloric material for low temperature operation, such as would be necessary for gas liquefaction. To broaden the scope, we can consider the manganites, which are a class of materials whose critical temperatures span from 10s of Kelvin to more than 350 K. As can be determined from the very comprehensive MCE review of these materials^3^, the median magnetic entropy change for a 5 T field change, independent of operating temperature, is about 8 J/kgK. This is about an order of magnitude larger than the transition metal based materials we describe here. As we are pioneering the study of these high entropy alloys for magnetocaloric applications, it is highly probable that refinement of the material, including the discovery of new compositions, will lead to improvements that can put HEAs on par with these and other materials.

Ultimately, the commercial viability of any refrigerant will depend on numerous factors often overlooked by basic science research. In the present case, cooling power per stable dollar is a critical advantage of transition metal systems over rare earths^29^. Other advantages include ductility, machinability, resistance to wear and corrosion, and reduced metal flammability. Since high entropy alloys address many of these issues, and do not suffer from the materials fatigue issues that limit the deployment of first order materials, it is clear that they have potential to serve as magnetic refrigerants. However, these systems have largely been unexplored simply because the parameter space occupied by 4, 5, and 6 element alloys is extremely vast. Since our results suggest the materials display mean field behavior, it may be possible for computational studies to explore this parameter space, thereby helping materials synthesizers to focus on certain compositions that have the most potential for near room temperature operation.

## Additional Information

**How to cite this article**: Belyea, D. D. *et al.* Tunable magnetocaloric effect in transition metal alloys. *Sci. Rep.* **5**, 15755; doi: 10.1038/srep15755 (2015).

## References

[b1] Gschneidner K. A. & Pecharsky V. K. Magnetocaloric Materials. Annu. Rev. Mater. Sci. 30, 387–429 (2000).

[b2] Gschneidner Jr K. A., Pecharsky V. K. & Tsokol A. O. Recent developments in magnetocaloric materials. Rep. Prog. Phys. 68, 1479–1539 (2005).

[b3] Phan M.-H. & Yu S.-C. Review of the magnetocaloric effect in manganite materials. J. Magn. Magn. Mater. 308, 325–340 (2007).

[b4] Singh N. K. *et al.* Itinerant electron metamagnetism and magnetocaloric effect in *RCo*_2_-based *L*aves phase compounds. J. Magn. Magn. Mater. 317, 68–79 (2007).

[b5] Bruck E., Tegus O., Thanh D. C., Trung N. T. & Buschow K. A review on *Mn* based materials for magnetic refrigeration: Structure and properties. Int. J. Refrig. 31, 763–770 (2008).

[b6] Miller C. W., Belyea D. D. & Kirby B. J. Magnetocaloric effect in nanoscale thin films and heterostructures. J. Vac. Sci. Technol., A 32, 040802 (2014).

[b7] Pecharsky V. K. & Gschneidner K. A. Jr. Giant magnetocaloric effect in *Gd*_5_(*Si*_2_*Ge*_2_). Phys. Rev. Lett. 78, 4494–4497 (1997).

[b8] Pecharsky V. K. & Gschneidner K. A. Jr. Tunable magnetic regenerator alloys with a giant magnetocaloric effect for magnetic refrigeration from 20 to 290 K. Appl. Phys. Lett. 70, 3299–3301 (1997).

[b9] Jr. K. G. & Pecharsky V. Thirty years of near room temperature magnetic cooling: Where we are today and future prospects. Int. J. Refrig. 31, 945–961 (2008).

[b10] Wada H. & Tanabe Y. Giant magnetocaloric effect of *MnAs*_1−*x*_*Sb*_*x*_. Appl. Phys. Lett. 79, 3302–3304 (2001).

[b11] Tegus O., Bruck E., Buschow K. H. J. & de Boer F. R. Transition-metal-based magnetic refrigerants for room-temperature applications. Nature 415, 150–152 (2002). 11805828 10.1038/415150a

[b12] Yeh J. *et al.* Nanostructured high-entropy alloys with multiple principal elements: Novel alloy design concepts and outcomes. Adv. Eng. Mater. 6, 299–303 (2004).

[b13] Gludovatz B. *et al.* A fracture-resistant high-entropy alloy for cryogenic applications. Science 345, 1153–1158 (2014). 25190791 10.1126/science.1254581

[b14] Lucas M. S. *et al.* Magnetic and vibrational properties of high-entropy alloys. J. Appl. Phys. 109, 07E307 (2011).

[b15] Inoue A. High strength bulk amorphous alloys with low critical cooling rates (overview). Mater. Trans. JIM 36, 866–875 (1995).

[b16] Halder M., Yusuf S. & Nigam A. Magnetocaloric effect and its implementation in critical behavior study of *Mn*_4_*FeGe*_3−*x*_*Si*_*x*_ intermetallic compounds. J. Appl. Phys. 110, 113915 (2011).

[b17] Bingham N. S., Phan M. H., Srikanth H., Torija M. A. & Leighton C. Magnetocaloric effect and refrigerant capacity in charge-ordered manganites. J. Appl. Phys. 106, 023909 (2009).

[b18] McHenry M. E., Willard M. A. & Laughlin D. E. Amorphous and nanocrystalline materials for applications as soft magnets. Prog. Mater Sci. 44, 291–433 (1999).

[b19] Franco V., Conde A., Kuz'min M. D. & Romero-Enrique J. M. The magnetocaloric effect in materials with a second order phase transition: Are t_*c*_ and t_*peak*_ necessarily coincident? J. Appl. Phys. 105, 07A917 (2009).

[b20] Lucas M. S. *et al.* Thermomagnetic analysis of *FeCoCr*_*x*_*Ni* alloys: Magnetic entropy of high-entropy alloys. J. Appl. Phys. 113, 17A923 (2013).

[b21] Sawada H. Residual electron density study of chromium sesquioxide by crystal structure and scattering factor refinement. Mater. Res. Bull. 29, 239–245 (1994).

[b22] Liu J., Gottschall T., Skokov K. P., Moore J. D. & Gutfleisch O. Giant magnetocaloric effect driven by structural transitions. Nature Mater. 11, 620–626 (2012). 22635044 10.1038/nmat3334

[b23] Pecharsky V. K. & Gschneidner J. K. A. Some common misconceptions concerning magnetic refrigerant materials. J. Appl. Phys. 90, 4614–4622 (2001).

[b24] Provenzano V., Shapiro A. J. & Shull R. D. Reduction of hysteresis losses in the magnetic refrigerant *Gd*_5_*Ge*_2_*Si*_2_ by the addition of iron. Nature 429, 853–857 (2004). 15215859 10.1038/nature02657

[b25] Sandeman K. G. Magnetocaloric materials: The search for new systems. Scripta Materialia 67, 566–571 (2012).

[b26] Franco V. & Conde A. Scaling laws for the magnetocaloric effect in second order phase transitions: From physics to applications for the characterization of materials. Int. J. Refrig. 33, 465–473 (2010).

[b27] Franco V., Conde A., Pecharsky V. & Gschneidner K. Jr. Field dependence of the magnetocaloric effect in *Gd* and (*Er*_1−*x*_*Dy*_*x*_)*Al*_2_: Does a universal curve exist? Europhys. Lett. 79, 47009 (2007).

[b28] Franco V., Caballero-Flores R., Conde A., Dong Q. & Zhang H. The influence of a minority magnetic phase on the field dependence of the magnetocaloric effect. J. Magn. Magn. Mater. 321, 1115–1120 (2009).

[b29] Ucar H., Ipus J., Franco V., McHenry M. & Laughlin D. Overview of amorphous and nanocrystalline magnetocaloric materials operating near room temperature. JOM 64, 782–788 (2012).

